# Elongation and plasmonic activity of embedded metal nanoparticles following heavy ion irradiation†

**DOI:** 10.1039/d3ra00573a

**Published:** 2023-02-16

**Authors:** Spyridon Korkos, Kai Arstila, Kosti Tapio, Sami Kinnunen, J. Jussi Toppari, Timo Sajavaara

**Affiliations:** a Accelerator Laboratory, Department of Physics, University of Jyväskylä P. O. Box 35 FI-40014 Jyvaskyla Finland spyridon.s.korkos@jyu.fi kai.arstila@jyu.fi sami.a.kinnunen@jyu.fi timo.sajavaara@jyu.fi; b Nanoscience Center and Department of Physics, University of Jyväskylä P. O. Box 35 FI-40014 Jyvaskyla Finland kosti.t.o.tapio@jyu.fi j.jussi.toppari@jyu.fi

## Abstract

Shape modification of embedded nanoparticles by swift heavy ion (SHI) irradiation is an effective way to produce nanostructures with controlled size, shape, and orientation. In this study, randomly oriented gold nanorods embedded in SiO_2_ are shown to re-orient along the ion beam direction. The degree of orientation depends on the irradiation conditions and the nanorod's initial size. SHI irradiation was also applied to modify spherical metallic nanoparticles embedded in Al_2_O_3_. The results showed that they elongate due to the irradiation comparably to those embedded in SiO_2_. Metallic nanostructures embedded in dielectric matrices can exhibit localized surface plasmon (LSP) modes. The elongated nanoparticles investigated by means of dark-field spectroscopy showed two discrete peaks which correspond to longitudinal and transverse modes.

## Abstract 2

Shape modification of embedded nanoparticles by swift heavy ion (SHI) irradiation is an effective way to produce nanostructures with controlled size, shape, and orientation.

## Graphical abstract

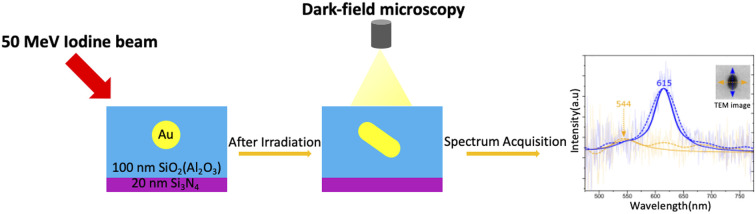

## 1 Introduction

The optical properties of embedded metallic nanostructures in dielectric materials are of particular interest because they originate from the localized surface plasmon (LSP) modes.^1–7^ These modes show a strong extinction peak at the localized surface plasmon resonance (LSPR) frequency. The LSPR peak arises from the collective oscillations of the conduction electrons confined within the volume of the nanoparticle. Moreover, at the resonance the electric field near the nanoparticle can be greatly enhanced due to the excess electric near-field created by the oscillating electrons of the plasmonic excitation. This near-field enhancement can lead to numerous applications, such as surface enhanced Raman spectroscopy,^8^ fluorescent or emission enhancement,^2,9^ photovoltaic and photocatalytic devices,^10^ optical waveguides,^11^ and nanolasers.^12^ Since these phenomena depend mainly on the surrounding material and the material and shape of nanostructures, the nanofabrication of this metal-dielectric system is of the greatest interest.

The most challenging part is the fabrication of the nanostructures as the optical response can depend not only on their material, shape, and size but their orientation as well. Instead of the standard nanofabrication techniques (colloidal chemistry, electron beam lithography or ion implantation), swift heavy ion (SHI) irradiation can modify the shape of embedded nanostructures (gold nanostructures embedded in SiO_2_ is the most studied system) in order to produce perfectly aligned nanorods along the ion beam direction.^13–21^ Especially, colloidal chemistry permits the synthesis of metallic nanoparticles of various shapes in liquid solutions, but with random orientations after the dispersion on a surface. In the case of electron beam lithography, the creation of well separated and aligned nanorods is time consuming for larger areas. During this process, known as ion beam shaping, the ion first forms a track in the host matrix leading to a decrease of density in the matrix (under densification). When the energy from the ions is transferred to the electrons of the metallic nanoparticle, the energy diffuses rapidly outward to the surface. As the energy reaches the metal/matrix boundary and is transferred to the electrons of the surrounding matrix, the temperature increases at the boundary due to the stronger electron–phonon coupling of the surrounding dielectric matrix. However, the surrounding matrix prevents the energy diffusion because of the lower thermal conductivity. Consequently, the electronic energy is transformed to heat and diffuses back toward the center of the nanoparticle resulting in full or partial melting.^17^ Finally, the molten metal flows into the track leading in elongation after cooling and recrystallization.^19,22^ This is not the only candidate mechanism as ion hammering effect has been proposed as well.^23–25^ However, Amekura *et al.*^26^ have shown that the latter mechanism is inconsistent with the experimental results.

The anisotropic nanorod shape offers two discrete LSPR peaks corresponding to longitudinal and transverse oscillation of the electrons. A suitable way to detect these two modes in a single nanoparticle is by applying dark-field optical microscopic spectroscopy. Dark-field spectroscopy is a well-established scattering technique for measuring the LSPR spectrum of single nanoparticles.^27–29^ The use of a broadband non-coherent low intensity light source does not cause deformation of the particles during imaging, which could lead to different plasmonic properties. However, the limitation on detection arise from the fact that scattering cross-section depends mainly on nanoparticle radius^30^ and as a result, nanoparticles with diameters less than 30 nm are difficult to detect and hence cannot be separated from their surrounding environment even with the very sensitive setup utilized in this work.^31^

Earlier studies have shown that the elongation of the nanoparticles along the ion beam direction strongly depends on the initial size of the nanoparticles and the applied fluence.^15,17,22,32^ However, this dependence on the initial size has not been extended for nanoparticles embedded to other material matrices apart from SiO_2_ in contrast to the great variety of metallic species used.^4,14–17,21,33,34^ Moreover, there is a shortage of studies regarding the nanoparticles shape other than spherical.

In a previous study,^35^ we showed that SHI irradiated gold nanorods, which are embedded in SiO_2_ and laid in a plane with a 45° angle to the ion beam direction, re-oriented to align with the beam. However, the impact of different fluences on the nanorods was not investigated experimentally. In addition, Atomic Layer Deposition (ALD) was used to deposit SiO_2_ which offered greater elongation in nanoparticles embedded inside that than in Plasma-Enhanced Chemical Vapor Deposition (PECVD) SiO_2_.^32^ The ALD can be used easily for the deposition of other films as well, such as amorphous Al_2_O_3_, TiO_2_, ZnO, *etc.* These have not been investigated in connection with SHI irradiation of embedded nanoparticles.

In this study, Au and Ag nanoparticles of various size and shape were embedded in amorphous SiO_2_ and amorphous Al_2_O_3_ deposited on top of Si_3_N_4_ TEM windows grid. The samples were irradiated by energetic ions using various fluences. Apart from chemically synthesized spherical nanoparticles, focused ion beam (FIB) lithography was used as well to create nanorods prior to irradiation. Within this technique, the desired nanostructure shape, size, and orientation on the surface of a film can be created using the capabilities of a Helium Ion Microscope (HIM) equipped with a Ne^+^ beam. By this way, the modification of nanorods inside SiO_2_ and spherical nanoparticles inside Al_2_O_3_ following heavy ion irradiation was investigated in detail depending on their initial size and fluence. The nanoplasmonic activity of irradiated nanoparticles was studied by applying dark-field spectroscopy to collect the spectra from individual nanoparticles. The obtained new information about the elongation process can be used to design and conduct new experiments and devices utilizing this method.

## 2 Experimental section

### 2.1 Sample preparation

Two types of samples were fabricated depending on the different matrix material and the shape of nanoparticles embedded inside them. Both were created on top of a TEM grid with nine windows of 20 nm thick Si_3_N_4_.

#### 2.1.1 Nanorods in SiO_2_

During the fabrication of the first type of the samples, 50 nm of ALD SiO_2_ were initially deposited. Then, Au nanorods made by HIM nanolithography were fabricated on top. In order to embed the nanorods inside SiO_2_, another 50 nm of SiO_2_ was deposited by ALD.^32^

**Fig. 1:**
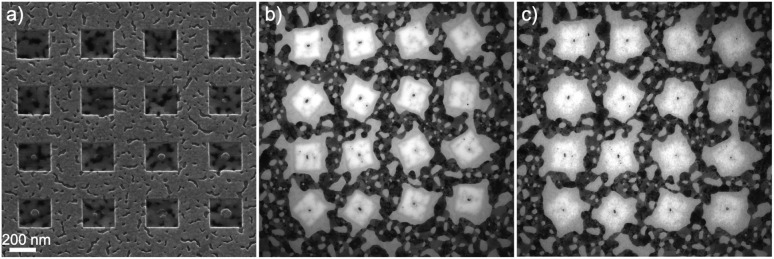
(a) HIM image after the patterning of nanostructures with a Ne^+^ beam, each rectangular area has slightly different processing parameters, (b) TEM image of patterned nanostructures different from (a) after embedding them in SiO_2_ prior to irradiation, and (c) TEM image of the same nanostructures as in (b) after the irradiation. The scale is the same in all the three images.

The fabrication of Au nanorods was made as following: a thin layer of gold (15–20 nm thickness) was first deposited using Ultra High Vacuum (UHV) evaporator. The next step was the milling of a selected area by 30 keV Ne^+^ beam in HIM. The Ne^+^ beam was used to mill away the gold layer from a rectangular area leaving to the center a nanorod of desired size and orientation as shown in Fig. 1. One of the three dimensions of the nanorods made by nanolithography was limited to 15–20 nm due to the original Au film thickness.

Additionally, samples with chemically synthesized gold nanorods embedded in SiO_2_ deposited by Plasma-Enhanced Chemical Vapor Deposition (PECVD) were created as described in ref. 35. On top of a TEM grid, 50 nm of PECVD SiO_2_ was firstly deposited and then chemically synthesized gold nanorods were dispersed on top. Finally, another 50 nm of PECVD SiO_2_ was deposited in order to encapsulate the nanorods inside SiO_2_.

#### 2.1.2 Spherical nanoparticles in Al_2_O_3_

The second type of the samples consisted of 50 nm of Al_2_O_3_ deposited by ALD on which chemically synthesized spherical Au (5–60 nm diameter) and Ag (10–25 nm diameter) nanoparticles were dispersed on top. The embedding of the nanoparticles was achieved by depositing another 50 nm of Al_2_O_3_.

#### 2.1.3 Thin film deposition and characterization

A Beneq TFS 200 cross flow reactor was used to deposit the thin films by ALD working at 100–200 Pa base pressure during the deposition. The depositions temperature was 200 °C. Nitrogen from Inmatec PN 1150 nitrogen generator (99.999% purity) was used as a carrier gas as well as for purging between the precursor pulses. For the deposition of SiO_2_,^32,36^ (3-aminopropyl)-triethoxysilane (APTES) (Sigma Aldrich, 99%), deionized water, and O_3_ were used as precursors. The growth of 50 nm SiO_2_ corresponded to 1250 cycles (36 h). For the deposition of Al_2_O_3_, trimethylaluminium (TMA) (Strem Chemicals, >98%) and deionised water were used as precursors.^37,38^ In order to deposit 50 nm Al_2_O_3_, 460 cycles (50 min) were applied.

For the deposition of SiO_2_ at 200 °C by PECVD, a Plasmalab80Plus (Oxford Instruments) machine was utilized using silane (SiH_4_ in Ar) and nitrous oxide (N_2_O) as precursors.^32,39^ The working pressure during the deposition was 133 Pa and the chamber was purged with N_2_ for 2 min before the deposition. The growth rate of the deposited film was approximately 50 nm min^−1^.

The properties of the grown films were investigated from films grown on Si substrate. The thicknesses and the refractive indexes of the deposited films were measured by spectroscopic ellipsometry (SOPRA GES 5E) equipped with a Xe lamp (75 W). The measured total thickness of the SiO_2_ films was 107.5 ± 0.5 nm, while Al_2_O_3_ film had 100.4 ± 0.6 nm thickness. Their refractive indexes were measured to be 1.52 and 1.66, respectively. Detailed composition analysis performed by Time-of-Flight Elastic Recoil Detection Analysis (ToF-ERDA)^40^ for films deposited using identical process is found in ref. 32 for SiO_2_ and ref. 38 for Al_2_O_3_. ALD-SiO_2_ and PECVD-SiO_2_ films are nearly stoichiometric with O/Si elemental ratios 2.07 ± 0.02 and 2.06 ± 0.02, respectively. Except silicon and oxygen, the film contain a significant amount of hydrogen (6.5 and 7.6 at%, respectively) as well as negligible amounts of carbon and nitrogen (less than 1 at%). ALD-Al_2_O_3_ film is almost stoichiometric with elemental ratio O/Al = 1.56 ± 0.03 and the contained impurities are hydrogen (2.8 at%) and carbon (less than 0.5 at%).

### 2.2 Sample irradiation and imaging

The samples fabricated on TEM grid were irradiated with 50 MeV ^127^I^9+^ ions at the TAMIA 5 MV tandem accelerator at Helsinki accelerator laboratory (University of Helsinki) as described in ref. 32. The angle of incidence was 45° and several fluences from 5 × 10^13^ ions per cm^2^ to 2 × 10^14^ ions per cm^2^ were applied at room temperature. Since the samples had such a thickness allowing the TEM imaging (120 nm totally), they were imaged with a JEOL-JEM 1400 TEM operated at 120 kV before and after the irradiation in order to track changes in the same nanoparticles. The shown TEM images prior to irradiation were taken from the top without tilting the TEM stage. However, the TEM images of the same nanoparticles after the irradiation were taken with the electron beam direction perpendicular to the ion beam direction in order to acquire the full information from the nanoparticles.

### 2.3 Optical characterization after the irradiation

**Fig. 2:**
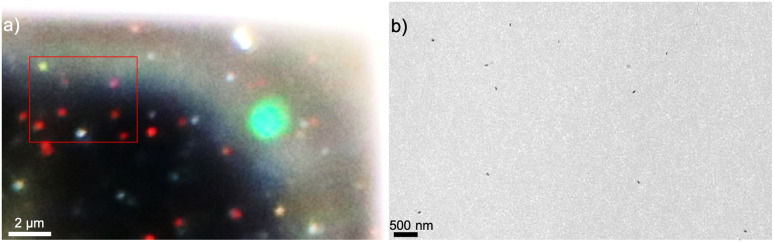
(a)A dark-field optical image from a sample containing nanostructures and (b) TEM image from the same sample containing the nanostructures located at the red rectangle in dark-field optical image. The bigger green circle denotes the fiber position and aperture size.

The optical characterization of the samples after the irradiation was made by dark-field optical microscopy.^41^ A brief description of the polarization dependent dark-field measurements is provided here (see ESI† for more details). An Olympus BX51TRF-microscope was used with Olympus 100 W halogen lamp. In order to investigate the plasmonic activity of the embedded nanostructures to determine LSPR peaks, the scattering spectra were measured at different polarization angles using an Olympus U-AN360-3 analyzer with Thorlabs linear polarizer (LPVISE200-A 2). The analyzer in combination with the polarizer was used to calibrate the polarization angle of the analyzer in respect to the sample surface. The scattered light from each individual nanostructure was collected by an optical fiber (core size 300 μm) placed to the image plane of the selected area. The spectra were recorded by a spectrograph (Princeton Instruments SP2150 (Acton)) equipped with a charge-coupled device (CCD) camera (Andor iVac DR-324B-FI), which was controlled using Andor Solis (version 4.18) software. Fig. 2 shows the same area with nanoparticles imaged both by TEM and dark-field optical microscopy.

The Finite Difference Time Domain (FDTD) simulations of the embedded and elongated nanoparticles were done using FDTD method in Ansys Lumerical software (FDTD, 2021 version 8.26.2834).^42^ During the simulations, we used two models to simulate the plasmonic scattering of the embbeded nanoparticle. Initially, we considered a model, where nanoparticles are surrounded by 100 nm thick medium (silicon dioxide or aluminum oxide) on top of a 20 nm thick silicon nitride window. There is an oil layer on top of the medium and air under the silicon nitride window. The incident light was injected from the oil side and characterized using scattering monitor. Polarization angles along the primary axis (transverse and longitudinal axis) were considered and the refractive indexes of 1.52 and 1.66 were used for SiO_2_ and Al_2_O_3_, respectively. Additionally, a simplified, infinite medium model was tested, where the particles were placed in an infinite medium and the refractive index of the medium was altered to match the measured and simulated LSPR peaks (see Fig. S2 and S3 in the ESI†). In this case, the refractive indexes of 1.38 and 1.55 for SiO_2_ and Al_2_O_3_ matched the two data.

## 3 Results and discussion

### 3.1 Irradiated Au nanorods embedded in SiO_2_

**Fig. 3:**
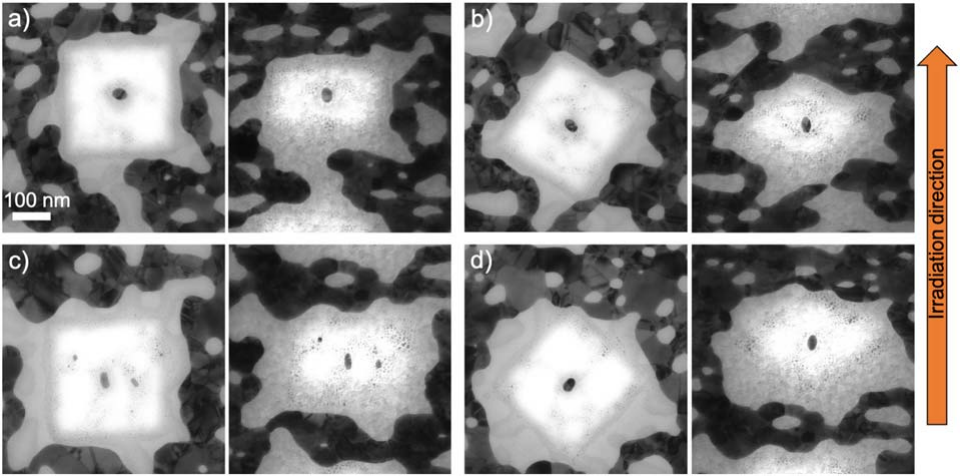
TEM images of fabricated nanorods with different orientations embedded in 100 nm ALD-SiO_2_ and irradiated with 50 MeV ^127^I at 5 × 10^13^ ions per cm^2^ before (left) and after (right) the irradiation. The TEM images prior to irradiation were taken from the top without tilting the TEM stage, while after the irradiation they were taken with the electron beam direction perpendicular to the ion beam direction. (a) Length = 39.2 nm and diameter = 29.5 nm, (b) length = 36.7 nm and diameter = 21.3 nm, (c) length = 38.6 nm and diameter = 20.9 nm, and (d) length = 36.4 nm and diameter = 26.5 nm. These nanorods re-oriented along the ion beam direction after the irradiation.

**Fig. 4:**
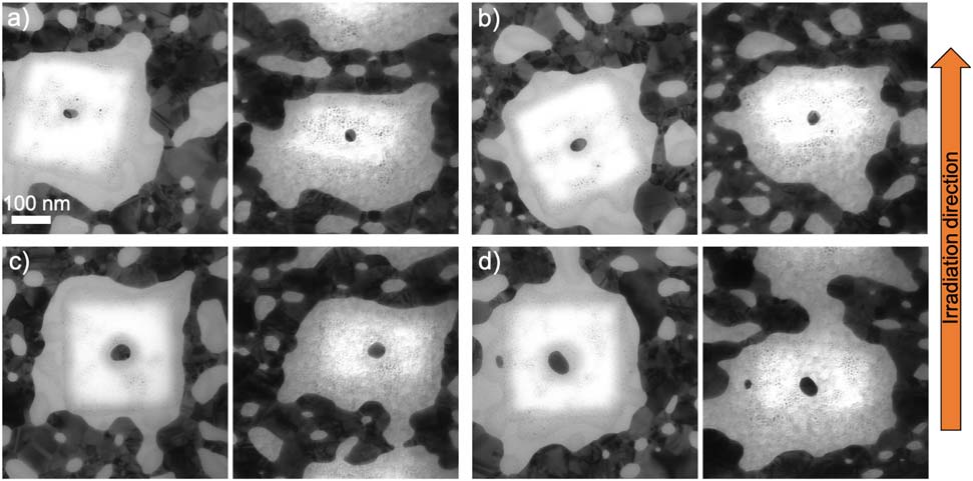
TEM images of fabricated nanorods with different orientations embedded in 100 nm ALD-SiO_2_ and irradiated with 50 MeV ^127^I at 5 × 10^13^ ions per cm^2^ before (left) and after (right) the irradiation. (a) Length = 35.9 nm and diameter = 24.0 nm, (b) length = 42.1 nm and diameter = 29.9 nm, (c) length = 51.9 nm and diameter = 37.1 nm, and (d) length = 58.2 nm and diameter = 36.0 nm. These nanorods did not manage to re-orient along the ion beam direction.

Three fluences were used for the irradiation of the fabricated nanorods embedded in ALD-SiO_2_. After 5 × 10^13^ ions per cm^2^ fluence, the shape changes were observed to depend on the size of the nanorods. Fig. 3 and 4 present nanorods of different sizes and orientations, before and after the irradiation. The nanorods in Fig. 3 were reshaped and elongated along the ion beam direction, while Fig. 4 presents nanorods, which were not elongated along the beam, but only slightly reshaped. In Fig. 4a and b, the nanorods have already started shrinking in the dimension perpendicular to the ion beam direction and elongating along the ion beam direction, but the applied fluence was not high enough to fully elongate them. Instead they turned to spheroids. The same effect can be observed as well in Fig. 4c in which the nanorod has started to incrementally reshape before its transformation to spheroid. In Fig. 4d, there is no change in the shape of this bigger nanorod comparing to others, but it is reduced in size due to the ion beam irradiation.

**Fig. 5:**
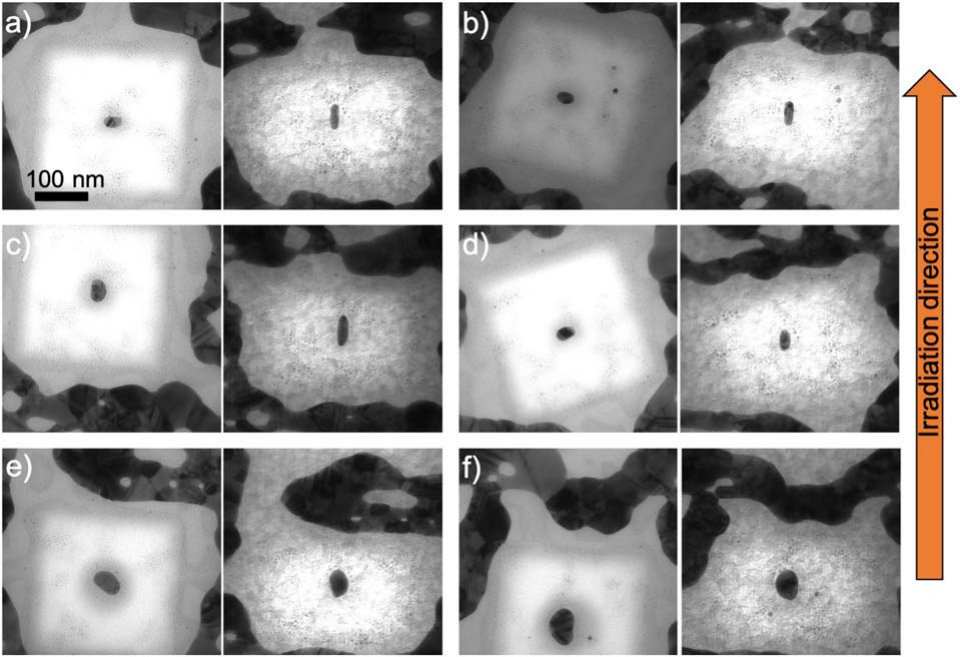
TEM images of fabricated nanorods with different orientations embedded in 100 nm ALD-SiO_2_ and irradiated with 50 MeV ^127^I at 10^14^ ions per cm^2^ before (left) and after (right) the irradiation. (a) Length = 34.0 nm and diameter = 19.9 nm, (b) length = 32.0 nm and diameter = 22.3 nm, (c) length = 42.6 nm and diameter = 23.4 nm and (d) length = 34.0 nm and diameter = 19.9 nm, (e) length = 50.4 nm and diameter = 31.2 nm, and (f) length = 70.4 nm and diameter = 42.3 nm.

After 10^14^ ions per cm^2^ fluence, the same reshaping effect occurred, *i.e.* nanorods with different orientation aligned along the ion beam direction (Fig. 5). The smallest nanorods elongate more than lower fluence, while bigger nanorods of the similar size as in Fig. 4c and d managed to also elongate along the ion beam direction (Fig. 5e). However, slightly bigger nanorod in Fig. 5f does not elongate along the ion beam direction.

**Fig. 6:**
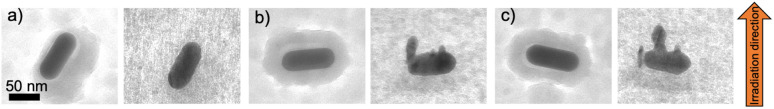
TEM images of chemically synthesized nanorods embedded in 100 nm PECVD SiO_2_ and irradiated with 50 MeV ^127^I at 10^14^ ions per cm^2^ before (left) and after (right) the irradiation. (a) Length = 86.0 nm and diameter = 35.0 nm, (b) length = 81.5 nm and diameter = 32.4 nm, and (c) length = 82.2 nm and diameter = 33.0 nm.

Chemically synthesized nanorods of similar size as in Fig. 5f and embedded in PECVD SiO_2_ were irradiated at 10^14^ ions per cm^2^ fluence as well. Some nanorods still cannot elongate (Fig. 6a), but the formation of random protrusions/spikes on several nanorods was observed as well (Fig. 6b and c).

**Fig. 7:**
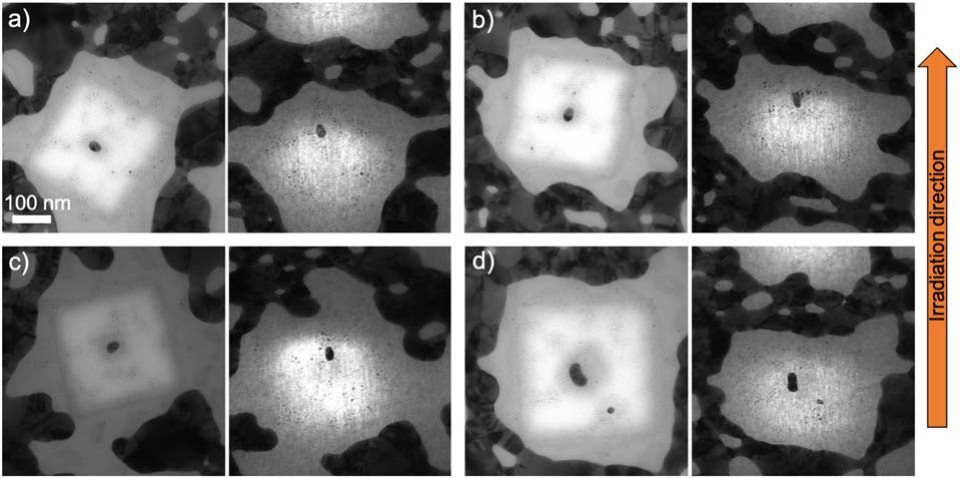
TEM images of fabricated nanorods with different orientations embedded in 100 nm ALD-SiO_2_ and irradiated with 50 MeV ^127^I at 2 × 10^14^ ions per cm^2^ before (left) and after (right) the irradiation. (a) Length = 33.2 nm and diameter = 22.6 nm, (b) length = 37.1 nm and diameter = 25.1 nm, (c) length = 35.0 nm and diameter = 24.4 nm, and (d) length = 62.8 nm and diameter = 29.2 nm before (left) and after (right) irradiation.

After the highest applied fluence (2 × 10^14^ ions per cm^2^), the smallest nanorods elongated along the ion beam direction (Fig. 7a–c) but not more than after lower fluences. The high irradiation fluence prevents them from greater elongation and forces them to shrink. This happens because of the size of a nanoparticle which is a basic parameter whether it will keep growing or starting disintegrate after significant ion fluence. The fact that the third dimension of the nanorod is always originally close to 15 nm can lead to the disintegration since nanoparticles below that critical size can start to disintegrate as shown in ref. 32. A bigger nanorod of the similar size as in Fig. 4c and d elongates along the ion beam direction in this highest fluence (Fig. 7d).

The modification of the nanorods can be explained^35^ as small incremental shape changes caused by highly energetic ions resulting in growth in the beam direction and loss of length in the other directions. The first ion impacts form protrusions onto the nanorods (as shown in Fig. 4c) and when more impacts take place, they transform the nanorod to a spheroid (as shown in Fig. 4a and b) and then to a nanorod aligned with the beam (as shown in Fig. 3). However, even if the theoretical studies have shown that large nanorods cannot be modified because of the interface effects,^43^ the formation of protrusions/spikes on them takes place along the ion beam direction at high enough fluences (≥10^14^ ions per cm^2^).

### 3.2 Irradiated nanoparticles embedded in ALD-Al_2_O_3_

**Fig. 8:**
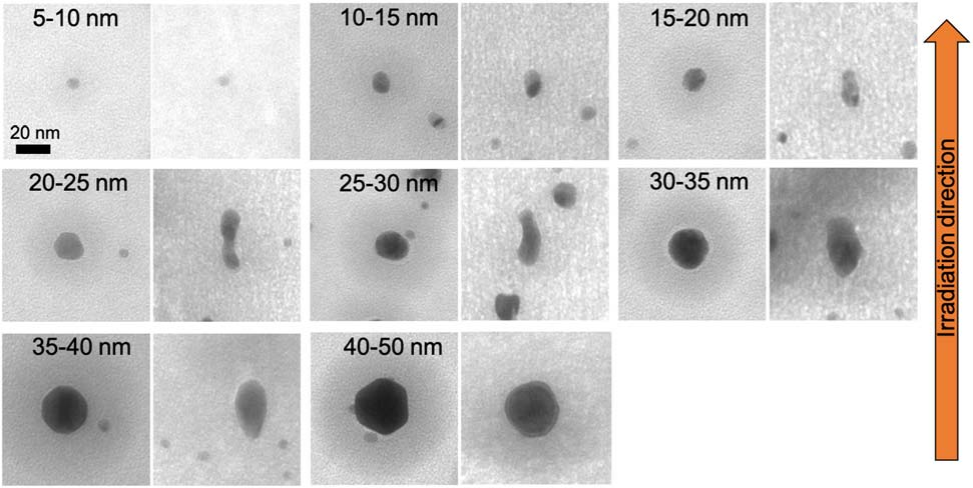
TEM images of elongated Au nanoparticles sandwiched between two 50 nm ALD-Al_2_O_3_ layers and irradiated with 50 MeV ^127^I at 5 × 10^13^ ions per cm^2^ before (left) and after (right) the irradiation.

**Fig. 9:**
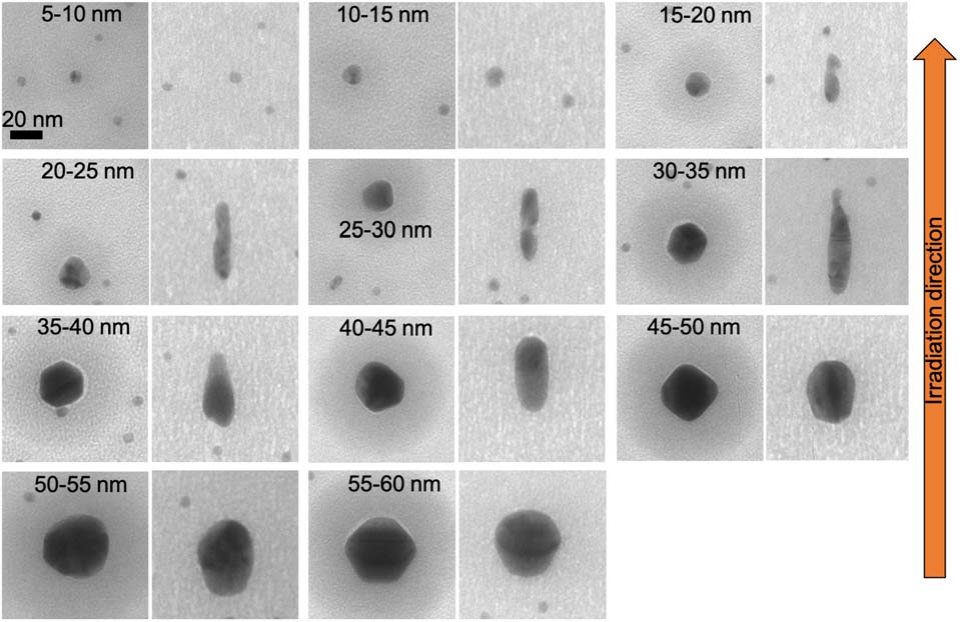
TEM images of elongated Au nanoparticles sandwiched between two 50 nm ALD-Al_2_O_3_ layers and irradiated with 50 MeV ^127^I at 2 × 10^14^ ions per cm^2^ before (left) and after (right) the irradiation.

**Fig. 10:**
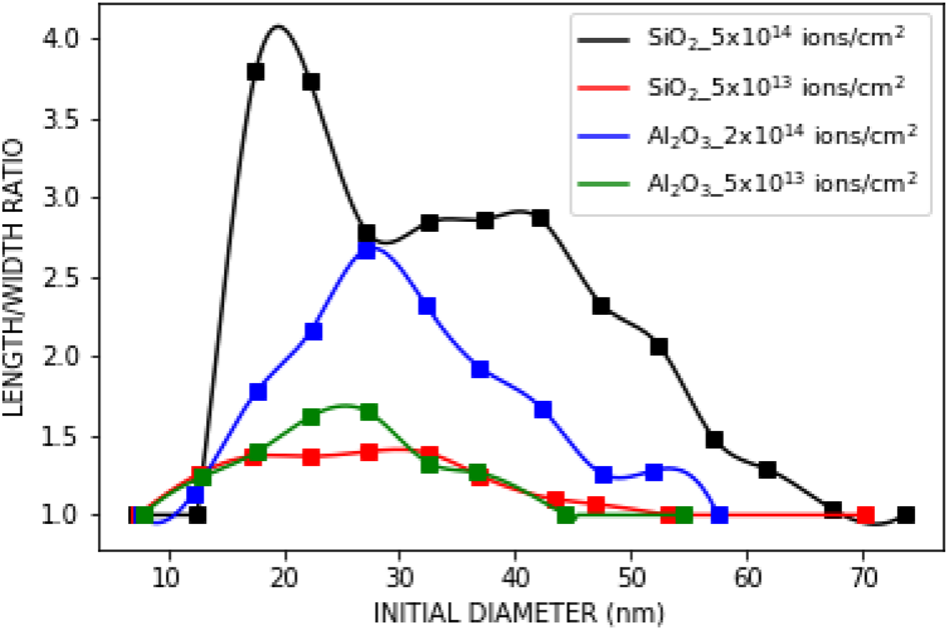
Comparison of the elongation ratio *vs.* initial diameter for the nanoparticles embedded in ALD-SiO_2_ (ref. 32) and ALD-Al_2_O_3_. The uncertainties are left out from the image for clarity.

Spherical Au and Ag nanoparticles embedded in ALD-Al_2_O_3_ were irradiated the same way as the SiO_2_ samples. In Fig. 8 and 9, the elongation of spherical Au nanoparticles is shown for 5 × 10^13^ and 2 × 10^14^ ions per cm^2^ fluences, respectively. As the fluence increases, the elongation ratio increases as well (Al_2_O_3_ curves in Fig. 10). For both fluences, the maximum elongation ratio is observed for nanoparticles of 25–30 nm diameter.

Before the irradiation, the nanoparticles (spherical and nanorods) are surrounded by a dark spot which indicates an area with a higher density.^32^ However, after the irradiation, these spots have been disappeared resulting in a more homogeneous matrix.

Comparing these results with ALD-SiO_2_ (Fig. 10) after 5 × 10^13^ ions per cm^2^ fluence,^32^ Al_2_O_3_ samples exhibit slightly higher elongation ratio than SiO_2_ samples only for initial size from 15 to 30 nm. At higher fluences, there cannot be absolute comparison since SiO_2_ samples are irradiated with a double fluence (5 × 10^14^ ions per cm^2^). With this difference in the fluence, for nanoparticles with initial size from 15 to 25 nm, the elongation is almost doubled if embedded in SiO_2_ compared to Al_2_O_3_. For the rest of the nanoparticles, the difference in ratio is smaller.

These results show that Au nanoparticles embedded in Al_2_O_3_ can sufficiently elongate with comparable ratio as in SiO_2_. According to Mota-Santiago *et al.*,^21^ the electron–phonon coupling and the thermal conductivity of the host matrix cause the different nanoparticles elongation between two host materials (SiO_2_ and Si_3_N_4_ in that case). On the one hand, the higher electron–phonon coupling of SiO_2_ (*g*_SiO_2__ = 1.25 × 10^19^ W m^−3^ K^−1^, *g*_Si_3_N_4__ = 0.52 × 10^19^ W m^−3^ K^−1^) implies greater energy transfer from the electronic subsystem to the lattice. On the other hand, the smaller thermal conductivity of SiO_2_ (*k*_SiO_2__ = 3 W_Al_2_O_3__ m^−1^ K^−1^, *k*_Si_3_N_4__ = 11 W m^−1^ K^−1^) leads to slower cooling resulting in more material flow into the ion track. Consequently, Au nanoparticles elongate more in SiO_2_ than in Si_3_N_4_. In our case, Al_2_O_3_ has lower electron–phonon coupling (*g* = 0.48 × 10^19^ W m^−2^ K^−1^) and higher thermal conductivity (*k*_Al_2_O_3__ = 30 W m^−1^ K^−1^) than SiO_2_. Nevertheless, there is no much difference in nanoparticles elongation ratio between them.

**Fig. 11:**
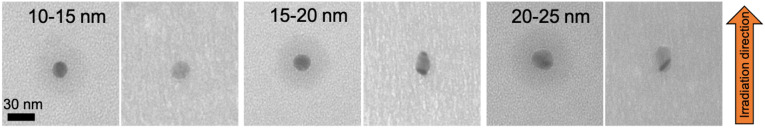
TEM images of elongated Ag nanoparticles sandwiched between two 50 nm ALD-Al_2_O_3_ layers irradiated with 50 MeV ^127^I at 2 × 10^14^ ions per cm^2^ before (left) and after (right) the irradiation.

Furthermore, Ag nanoparticles exhibited smaller elongation ratio than Au nanoparticles. As shown in Fig. 11, the Ag nanoparticles irradiated at 2 × 10^14^ ions per cm^2^ fluence achieve in average only half the elongation ratio compared to the Au nanoparticles at the same fluence. This is consistent with previous experiments in which Au nanoparticles embedded in SiO_2_ elongate more than Ag nanoparticles.^15,17^

### 3.3 Optical characterization of irradiated nanoparticles embedded in SiO_2_ and Al_2_O_3_

The determination of localized surface plasmon resonance (LSPR) peaks of the embedded nanostructures after the irradiation was achieved by dark-field optical microscopic spectroscopy which allowed to locate and measure the scattering spectra of individual nanostructures.^41,44^ Some limitations appeared during the experiments preventing the collection of spectra for each size of nanostructures. The distance between two nanostructures as well as the size of the fiber spot (approximately 1–2 μm diameter) made the distinction between the nanoparticles many times difficult. Moreover, only elongated nanoparticles with width more than 30 nm could be imaged.^31,45,46^ The reason is that the absorption is dominating for sizes less than 30 nm resulting in weak scattering, which even in an ideal case could be lost in the background scattering signal.^31^ In our case, the background scattering is increased further due to transparent, uneven substrate with several interfaces that scatter light. In addition, the fact that the nanoparticles are embedded inside a material and not lying on a surface worsens the resolution.

**Fig. 12:**
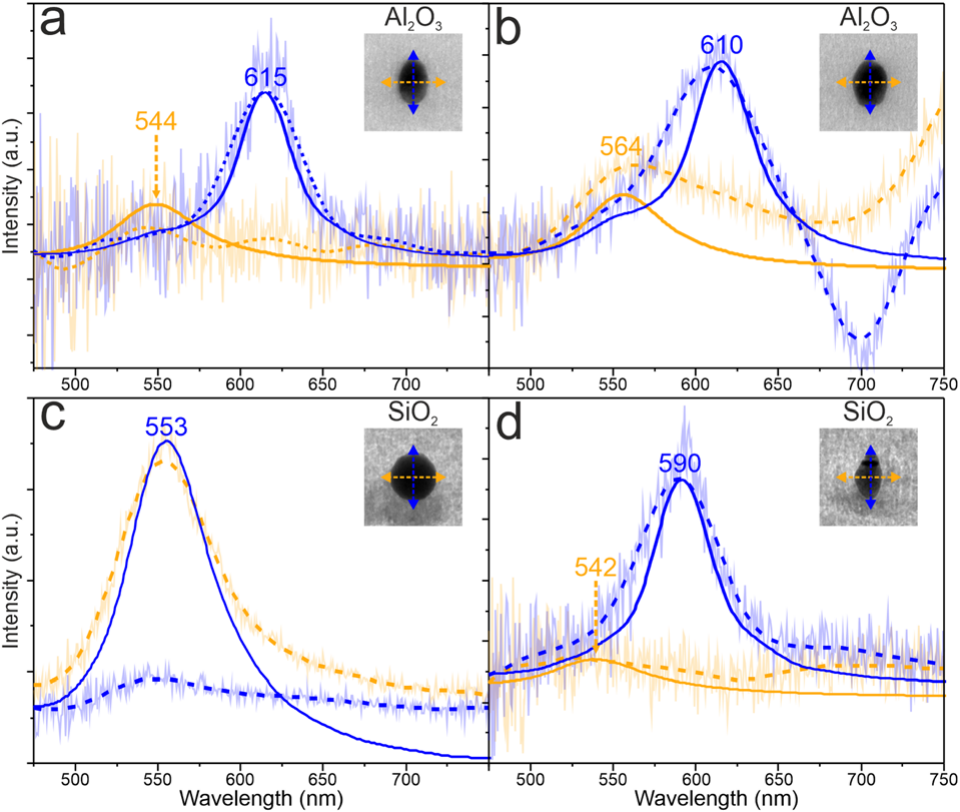
Dark-field scattering spectra of two elongated nanoparticles embedded in Al_2_O_3_ (a and b), one spherical nanoparticle embedded in SiO_2_ (c) and one elongated nanoparticle embedded in SiO_2_ (d). The solid line is the result from the FDTD simulation and the dashed line the averaged measurement, with original data shown as a lighter line. The blue and the yellow spectra correspond to the longitudinal and transverse modes, respectively. The inset TEM images show the measured particles with LSP polarizations marked. The size of the TEM images is 110 nm × 110 nm. The lengths and the widths of the particles in (a) and (b) are 51.3 nm and 32.5 nm and 51.9 nm and 39.9 nm, respectively. The diameter of the particle in (c) is 53.6 nm and the length and the width of the particle in (d) are 52.5 nm and 31.9 nm, respectively.

The spectra with the corresponding nanostructures are shown in Fig. 12. To determine the origin of the LSPR peaks, we ran FDTD simulations using Ansys Lumerical. The simulated spectra were extracted using the simplified model to reduce the simulation time. The spherical nanoparticle in Fig. 12c exhibits one (dipolar) plasmon mode, while the elongated nanoparticles in Fig. 12a, b and d exhibit two plasmon modes. The main peak corresponds to the longitudinal mode of the elongated nanostructure while the second, smaller peak corresponds to the transverse mode. The splits between the two LSPR modes in the case of Al_2_O_3_ are 71 nm and 46 nm in the Fig. 12a and b because of the different widths of the nanoparticles. For SiO_2_ the split is 48 nm in Fig. 12d. The transverse LSP mode in experimental spectrum of the elongated nanoparticle in Fig. 12d is very weak because the width of the nanoparticle reaches the limit of the size detection.^47^ The measured main LSPR peak is more red shifted in the case of Al_2_O_3_ compared to SiO_2_ due to higher refractive index of the surrounding medium.

The LSPR peaks of embedded nanoparticles inside SiO_2_, which have been irradiated with SHI, have already been investigated with the electron energy loss spectroscopy in scanning transmission electron microscope (STEM EELS).^4,7^ According to Kobylko *et al.*,^7^ the LSPR peak of an unirradiated spherical Au nanoparticle of around 22 nm diameter is located at 558 nm. Comparing this with our results, there is a slight shift of the LSPR peak to lower wavelength. In our case, the spherical nanoparticle is bigger and measured after the irradiation, which has caused structural changes in the surrounding material. Peña-Rodríguez *et al.*^48^ have demonstrated that the irradiation causes density changes in the surrounding material resulting to the change of the refractive index, which affects the LSPR peaks. In addition, an elongated nanoparticle of 61.6 nm length and 31.4 nm (aspect ratio 1.94) width has a longitudinal LSPR peak at 652 nm and a transverse peak at 532 nm. In our measurements, the elongated nanoparticle has length 52.5 nm and width 31.4 nm (aspect ratio 1.64). The transverse peak is located at 542 nm which differs slightly from Kobylko's result because the width in both cases is almost the same and there is a difference in irradiation conditions. On the other hand the longitudinal peak is located at 590 nm which differs significantly from Kobylko's because there is 9.1 nm difference in length.

## 4 Conclusions

In this work, we investigated the impact of SHI irradiation on embedded nanorods and showed that the irradiation modifies them in different ways depending on their initial size and applied fluence. As a result, the smallest ones with length less than 40 nm and diameter less than 30 nm are reshaped to nanorods aligned with the ion beam direction after any fluence applied. Nanorods with length between 40 and 50 nm and diameter between 30 and 40 nm irradiated with the lowest fluence only manage to turn to spheroids, while after higher fluences, they are reshaped along the ion beam direction. However, the largest ones with length between 70 and 86 nm and diameter between 30 and 45 nm either retain their shape or random protrusions/spikes are formed on them. Additionally, we studied the elongation of spherical gold and silver nanoparticles embedded in Al_2_O_3_. The results showed that gold nanoparticles can elongate similarly as in SiO_2_ even if they have different electron–phonon coupling and thermal conductivity. On the other hand, the silver nanoparticles elongate less than gold ones. Apart from these, the investigation of their nanoplasmonic activity showed that the elongated nanoparticles both in SiO_2_ and Al_2_O_3_ exhibit two discrete LSP modes (longitudinal and transverse) which make them potential candidates for photonics applications.

## Author contributions

Spyridon Korkos conceptualized the project, performed the experiments, analyzed and visualized the data, and wrote the original draft. Kai Arstila conceptualized the project and performed the experiments. Kosti Tapio performed the experiments, analyzed and visualized the data, and wrote the original draft. Sami Kinnunen performed the experiments. J. Jussi Toppari reviewed and edited the manuscript. Timo Sajavaara reviewed and edited the manuscript.

## Conflicts of interest

There are no conflicts to declare.

## Supplementary Material

RA-013-D3RA00573A-s001
