# Supplementary material for: Elongation and plasmonic activity of embedded metal nanoparticles following heavy ion irradiation
Source: RSC Adv. 2023 Feb 16;13(9):5851–8. doi: 10.1039/d3ra00573a (PMC9932636; doi:10.1039/d3ra00573a)
Supplement: RA-013-D3RA00573A-s001 [file RA-013-D3RA00573A-s001.pdf]

Electronic Supplementary Material (ESI) for RSC Advances.
This journal is © The Royal Society of Chemistry 2023

Supplementary Information: Elongation and plasmonic activity of embedded metal nanoparticles following heavy ion irradiation

Spyridon Korkos,[ab] Kai Arstila,[ab] Kosti Tapio,[b] Sami Kinnunen,[ab] J. Jussi Toppari,[b] and Timo Sajavaara[ab]

[a] Accelerator Laboratory, Department of Physics, University of Jyväskylä, P.O. Box 35, FI-40014 Jyvaskyla, Finland
[b] Nanoscience Center and Department of Physics, University of Jyväskylä, P.O. Box 35, FI-40014 Jyvaskyla, Finland

1 Polarization dependent dark-field measurements

Olympus BX51TRF microscope equipped with EOS 6D camera and coupled to Andor SP2150i spectrograph via a fiber (Thorlabs UM22-300-custom; core size, 300 μm) was used to measure the dark-field spectra [1, 2]. Andor IVAC DR-324-FI EMCCD camera was attached to the spectrograph and used to record the DF spectra. Olympus MPLANFL N 100x objective was utilized during the recording. IR-halogen lamp (100W, spectrum) was used to illuminate the samples in transmission mode. The microscope also included an analyzer (Olympus U-AN360-3) to filter the different light polarization coming from the sample.

Initially, the analyzer angle in respect to the optical camera view was calibrated: a thin metal wire was stretched and fixed on top of a polarizer (Thorlabs LPVISE200-A 2) and along the polarization axis of the polarizer. The polarizer was placed in the microscope and the wire was aligned along the vertical axis of the camera view. The analyzer was pushed in and we recorded the spectra originating from the polarizer while rotating the analyzer. The signal minimum (the perpendicular axis to the polarizer and the wire) and the maximum signal (the parallel axis to the polarizer) were noted and marked. We used these axes as the reference points, where the perpendicular axis was assigned as 0° and 180° due to 2-fold symmetry.

We removed the polarizer and placed a clean microscope slide in the microscope. The slide was washed first with detergent, then flushed with Milli-Q water and isopropanol, and finally dried using N_2 flow. A dark-field condenser (Olympus U-DCW) was placed underneath the slide and drop of oil was added to the condenser. The condenser and the slide were brought in contact so that there was thin oil layer between them. A small drop of oil was placed on top of the slide and excess oil was removed using a lens paper, until there was a thin layer of oil left. The sample was placed on top of the oil drop, so that the membrane window was touching the oil. If it seemed, that the sample was floating on top of the oil, we used a lens paper to remove oil around the sample, until the sample was immobile. During this process, reflected light (U-LH75XEAPO 75W Xenon lamp) was used to place the sample on the oil and this lamp was switched off during the measurements.

TEM reference images were used to find the correct window and the particles (Figure S1). The position of the fiber in the camera view was visualized by illuminating light from the spectrograph end using a green LED (Thorlabs M530L3). The polarization measurements were carried out by placing the fiber spot so that only the target particle was inside fiber spot, then the fiber was placed back to the spectrograph and desired polarization angle was selected (starting from the

angle 0°). The spectrum was recorded and we then selected an empty area next to the particle for the background (BG) measurements. The next polarization angle was selected and the spectra were recorded similarly for the particle and the background as before. This process was repeated for 0°, 45°, 90°, 135°, and 180° angles. After all the necessary particle spectra were recorded, we removed the sample and the condenser and recorded the lamp spectrum and the dark current (DC). The final particle intensity I was calculated from equation 1.

$$I = \frac{I_{AuNP} - I_{BG}}{I_{lamp} - I_{DC}}. \tag{1}$$

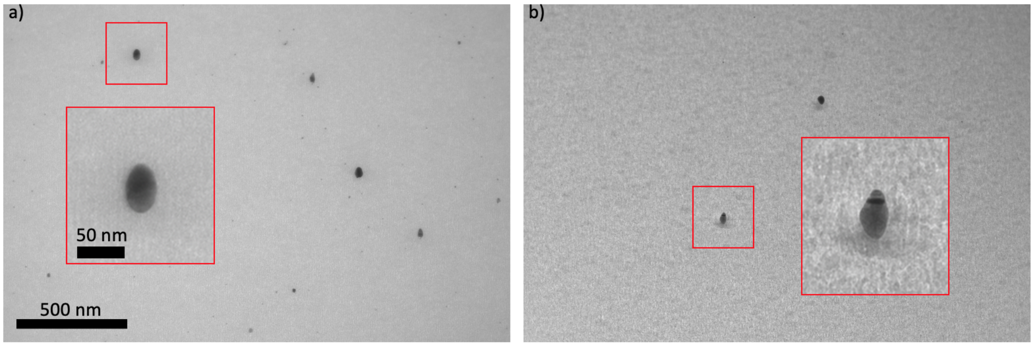

Figure S1: TEM images of nanoparticles embedded in (a) Al_2O_3, where the inset image in big red outline is the magnified image of the same nanoparticle in small red outline and (b) SiO_2, where similar images as in a) are presented.

2 FDTD simulations of elongated and embedded gold nanoparticles

The finite-difference time domain (FDTD) simulations were performed using Lumerical FDTD Solutions software 2021 version 8.26.2834. The simulations were done in two-fold: the initial model with the air-medium-silicon nitride interface was utilized, which was compared to infinite medium approximation that was then used to simulate all the results. The schematic view of the initial model is shown in Figure S2. The dimensions of the gold nanoparticles were taken from the TEM images such as in Figure S1, where it was assumed that the elongated nanoparticles are

ellipsoids with two equal length short axes and one long axis. The refractive index (R.I.) of Au was taken from Johnson and Christy [3].

Initially, we considered the air-oil-medium-silicon nitride-air interface, where the particles are embedded in the medium (either silicon dioxide SiO_2 or aluminum oxide Al_2O_3). We ran the simulation with the initial model for the particle shown in Figure S2 (the solid green curve), which matched the measured LSPR peaks of the same particle (the dashed blue and yellow curves). Here, the refractive indexes of SiO_2 and Al_2O_3 were 1.52 and 1.66, respectively. We then assumed an infinite medium approximation and fitted the simulated LSPR peaks to the experimental ones by changing the R.I. of the new infinite medium. For SiO_2 and Al_2O_3, the new refractive indexes were 1.38 and 1.55, respectively, and the data fits the measured data and the initial model (the solid blue and yellow curves in Figure S2). Here, the interpretation is that the new refractive indexes are averaged, distance weighted values between air, medium and silicon nitride, so that the system can be simplified to an infinite medium approximation and the simulation can be performed faster. The simulation for other particles (see Figure 12 in the main article) shows that the simulated peaks fit the data using this approximation. A mesh of 1 nm was used around the particles and an overall mesh of 20 nm was defined throughout the rest of simulated area. The refractive index of air was 1.00027. Different polarization angles were initially considered, but since our ellipsoid has only two main LSPR modes (the short axis and the long axis) only two polarization directions were considered corresponding to these modes (Figure S3), since the other polarization directions were superpositions of these two modes.

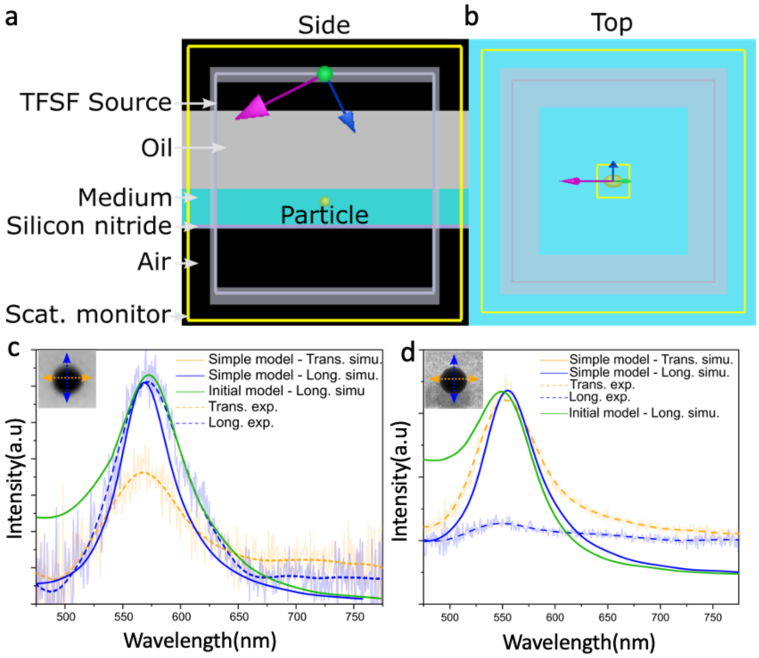

Figure S2: (a)-(b) The side and the top view of the initial FDTD model of the elongated and embedded particles in Al_2O_3 medium. (c) Comparison between the initial model and the simplified model for particle embedded in Al_2O_3. The solid green curve depicts the fit using the initial model, where as the solid blue and yellow curves depict the simplified model. The dashed curves show the corresponding measured data. (d) Comparison between the initial model and the simplified model for particle embedded in SiO_2. The solid green curve depicts the fit using the initial model, where as the solid blue and yellow curves depict the simplified model. The dashed curves show the corresponding measured data. The size of the TEM images is 110 nm×110 nm.

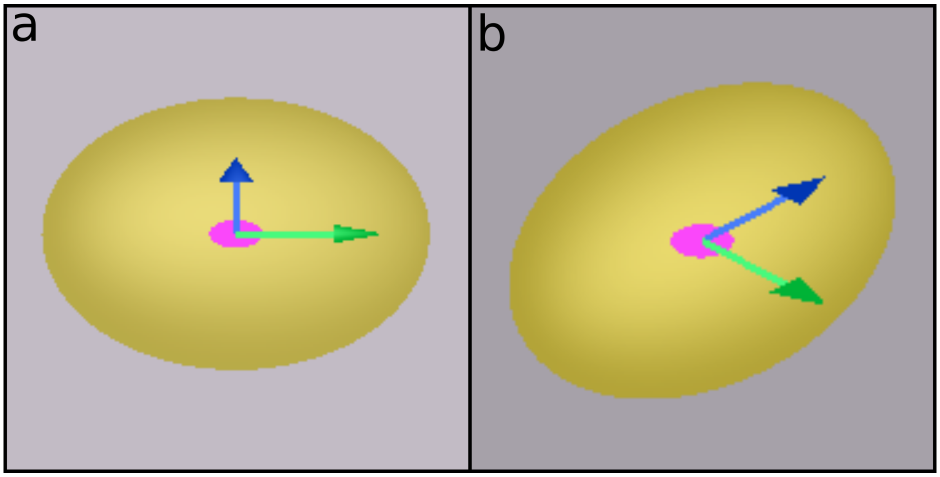

Figure S3: (a), (b) The two polarization angles considered in the simplified simulations.

References

[1] B. Shen, V. Linko, K. Tapio, S. Pikker, T. Lemma, A. Gopinath, K. V. Gothelf, M. A. Kostiainen and J. J. Toppari, *Science Advances*, 2018, **4**, eaap8978.

[2] K. Tapio, S. Dongkai, S. Auer, J. Tuppurainen, M. Ahlskog, V. Hytönen and J. Toppari, *Nanoscale*, 2018, **10**, 19297–19309.

[3] P. Johnson and R. Christy, *Physical Review B*, 1972, **6**, 4370–4379.
